# Neoadjuvant FOLFOXIRI in a Locally Advanced Right Colon Cancer Patient: A Case Report From a Limited‐Resource Setting

**DOI:** 10.1155/crom/5532415

**Published:** 2026-11-06

**Authors:** Osama Alazka, Rita Mohammad, Haya Alreem Saied, Hamza Abedallaa, Nader Abedallaa

**Affiliations:** ^1^ Department of Medical Oncology Latakia University Hospital Latakia Syria; ^2^ Cancer Research Center Latakia University Latakia Syria; ^3^ Faculty of Medicine Latakia University Latakia Syria

**Keywords:** FOLFOXIRI, locally advanced colon cancer, neoadjuvant therapy, right colon cancer, tumor regression grade

## Abstract

**Background:**

Although upfront surgery followed by adjuvant chemotherapy is the standard of care for locally advanced colon cancer (LACC), high rates of recurrence and surgical morbidity remain significant challenges in bulky T4 tumors. Neoadjuvant chemotherapy (NAC) is gaining traction to improve downstaging and resectability. Although doublet regimens (FOLFOX/CAPOX) are established, the use of the intensive triplet regimen FOLFOXIRI in the neoadjuvant setting for nonmetastatic LACC is less common because of toxicity concerns and limited clinical data.

**Case Presentation:**

A 55‐year‐old male presented with a bulky, obstructive, right‐sided colonic mass (cT4bN+M0). Given the high‐risk features and the need for significant cytoreduction in a resource‐limited setting where molecular profiling was unavailable, neoadjuvant FOLFOXIRI was initiated. After six cycles, the patient achieved an 85% reduction in tumor volume and normalization of tumor markers (CEA/CA19‐9). Despite experiencing manageable Grade 3 neutropenia and anorexia, he underwent successful R0 resection. Histopathology confirmed significant downstaging to ypT2pN0 (0/12), reflecting a near‐complete pathological response. Following four cycles of adjuvant CAPOX, the patient remains disease‐free at 1.5 years.

**Conclusion:**

Neoadjuvant FOLFOXIRI can achieve significant downstaging in bulky tumors and facilitates R0 resection in selected high‐risk LACC patients, even in resource‐limited settings. Despite requiring careful management of treatment toxicities, triplet NAC offers exploratory, hypothesis‐generating option for first colon cancer patients with T4 primary tumors, and can be further supported with future large clinical data.

## 1. Introduction

Colon cancer represents a major health burden, ranking third in incidence and second in cancer mortality worldwide [1]. Upfront surgical resection and adjuvant chemotherapy have been the standard of care in resectable, locally advanced colon cancer (LACC) [2]. However, the incidence of local recurrence and distant metastasis can be up to 35%, especially in high‐risk patients [3, 4], with a 5‐year disease‐free survival rate of 64.5% in high‐risk (Stage IIb/III) patients [4]. Primary wide surgical resection of bulky tumors (especially invading adjacent structures) carries high rates of morbidity and incomplete resection, albeit its necessity in the ambulatory settings [5].

Neoadjuvant chemotherapy (NAC) as a standard of care in rectal cancer, offers high effective rates of micro‐metastases eradication and downstaging of primary tumor, thus improving the outcomes of surgical resection. Based on this effective approach, the role of NAC in LACC has gained popularity over the last few years [4, 6]. However, concerns about delaying curative surgery or potential tumor complications, such as perforation or obstruction, were the main hurdles for application [7, 8]. More recently, clinical trials like the FOxTROT and OPTICAL trials reported that neoadjuvant therapy (using the doublet FOLFOX/CAPOX regimen) was safe and effective in tumor downstaging, providing similar analogues to neoadjuvant therapy in rectal cancer, in addition to improved 2‐year disease‐free survival rates compared with upfront surgery [9, 10]. Doublet NAC therapy was found highly effective in mismatch repair‐proficient (pMMR) tumors, but needed the addition of immune checkpoint inhibitors in mismatch repair‐deficient (dMMR) tumors to achieve significant treatment response [11, 12].

The FOLFOXIRI (triplet fluorouracil‐based protocol Fluorouracil, leucovorin, oxaliplatin, and irinotecan) has been widely applied in the metastatic setting in colon cancer, particularly in right‐sided tumors achieving high objective response rates. It is however applied in fit younger populations [13]. Nonetheless, the application of neoadjuvant FOLFOXIRI in nonmetastatic LACC is by all means an investigational approach [14]. Despite reporting major pathological response rates with triplet therapy, no prospective randomized control trials have compared doublet and triplet NAC in the setting of LACC [14, 15]. Furthermore, NCCN guidelines recommend primarily doublet regimens for respectable high‐risk disease [16].

We report the case of a patient with a bulky high‐risk Stage III T4b right‐sided colon cancer successfully treated with NAC FOLFOXIRI at a university cancer center in Syria (a resource‐limited setting), achieved significant tumor shrinkage and R0 resection omitting the need for wide adjacent organ resection, and sustained disease‐free survival at 1.5 years (18 months).

## 2. Case Presentation

A 55‐year‐old man presented with symptomatic anemia and nonspecific abdominal discomfort. His medical history included only uncomplicated cholecystectomy. Colonoscopy revealed a large, lobulated, subocclusive mass at the hepatic flexure. Endoscopic biopsy confirmed a moderately differentiated Grade 2 adenocarcinoma. CT scans of the chest, abdomen, and pelvis showed a heterogeneously enhancing mass measuring 88 × 120 × 127 mm infiltrating adjacent retroperitoneum (cT4b); estimated volume through ellipsoid equation: *V* = *π*/6 × 8.8 × 12 × 12.7 = 701.6 cm^3^ [17]. No distant metastases were reported in the baseline scan. [Figure 1A]. Baseline serum tumor markers were elevated (CEA: 24 ng/mL, CA19‐9: 34 U/mL; normal range: less than 5 ng/mL and less than 37 U/mL, respectively). Comprehensive molecular panel was not conducted due to local practice guidelines regarding the routine molecular testing of colon cancer (MMR status, KRAS/NRAS, and BRAF mutations), as it is required for metastatic patients, and the extremely high cost conducting these tests even so in Syria, where infrastructures and diagnostic reagent are hardly available.

**Figure 1:**
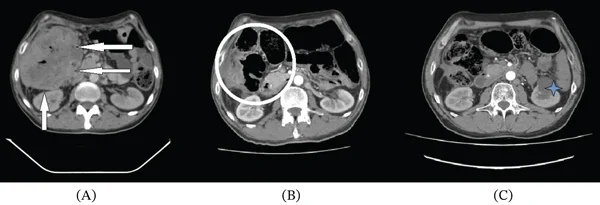
Perioperative computerized tomography scans (CT). (A) Transverse section showing baseline 88 × 120 × 127 mm colonic mass at the hepatic flexure (arrows). (B) Post three FOLFOXIRI cycles scan reveals 85% volumetric reduction in tumor size (circle). (C) One year follow‐up scan confirming absence of local recurrence or distant metastases (asterisk: incidental likely‐benign renal cyst).

A total of 85% of sporadic onset of colon cancer cases are pMMR, thus highly sensitive to chemotherapy [12]. In addition, the advanced size of the primary tumor and its location constitute a high‐risk disease requiring emergency surgical bowel diversion or multivisceral resection (MVR); the latter carries high rates of morbidity and mortality. The rationale of treatment decision was based on the need for achieving rapid tumor volume cytoreduction to lower the impending luminal obstruction and increase the probability to convert the disease′s treatment eligibility into a standard complete mesocolic excision. This decision was further supported by the patient′s age and good general status (ECOG 0). Neoadjuvant FOLFOXIRI was presumed the best option for this patient.

FOLFOXIRI comprised of biweekly schedule starting Day 1: oxaliplatin 85 mg/m2 IV infusion over 120 min, irinotecan 165 mg/m2 IV infusion over 90 min, calcium folinate 200 mg/m2 IV infusion over 120 min, 5‐fluorouracil 3200 mg/m2 IV infusion through peripheral catheter over 48 h. Standard antiemetic prophylaxis included ondansetron 8 mg and dexamethasone 12 mg. After 3 cycles (6 weeks), a restaging CT scan demonstrated an 85% volumetric tumor reduction (35 × 52 × 55 mm: estimated volume at 107.6 cm^3^), marked improvement in the luminal patency, and normalization of serum tumor markers (CEA: 3.75 ng/mL; CA19‐9: 11.9) [Figure 1B]. After the third cycle, the patient experienced Grade 3 neutropenia (absolute neutrophil count less than 0.7 × 10^9^/L) and Grade 3 anorexia. Treatment was halted for 5 days, and subcutaneous filgrastim 5 *μ*g/kg/day combined with enteral dietary nutritional supplementations until the complete resolution of hematological and gastrointestinal toxicities, that is, to Grade 0. Afterwards, chemotherapy was resumed with 100% planned dose intensity for three more cycles, supported with prophylactic subcutaneous filgrastim on Days 3 through 5 of each cycle. No further similar adverse events occurred or any need for hospitalization.

Elective open right hemicolectomy with complete mesocolic excision (CME) and central vascular ligation (D3 lymphadenectomy) was performed after the completion of the sixth cycle (3 months total therapy). Surgical report mentioned that during intraoperative exploration, the tumor had resided significantly from the retroperitoneum leaving a clear fibrotic cleavage plane. The surgeon was able to avoid multivisceral en bloc organ resection. Histopathological results confirmed a R0 resection with free surgical margins, 0/12 resected lymph nodes involved (ypT2, pN0, and cM0). Thus achieving a tumor regression Grade 1 (near‐complete pathological response) according to the modified Ryan/AJCC eighth edition criteria [18]. Postoperative serum tumor markers remained normal (CEA: 0.2 ng/mL; CA19‐9: 8.2 U/mL).

Treatment then proceeded to 4 cycles of adjuvant CAPOX (every 21 days, Days 1–14; oxaliplatin 135 mg/m^2^ IV infusion over 120 min on Day 1, capecitabine 2000 mg/m^2^/day orally divided in two doses for Days 1–14). Post‐adjuvant scans and tests were normal. Surveillance protocol consisted of clinical examination, serum tumor markers every 3 weeks, chest/abdomen/pelvis CT scan every 5 months. Colonoscopy at 1 year after surgery reported healthy, patent ileocolic anastomosis. At 1.5 years (18 months), the patient remains in complete clinical, biochemical, and radiological remission [Figure 1C].

## 3. Discussion

Neoadjuvant approach in LACC, as with rectal cancer, aims for a downstaging of the bulky primary tumors, enhance efficacy in early micrometastases eradication, and reduce surgical morbidity and mortality [2, 4]. The application of doublet chemotherapy (FOLFOX/CAPOX) is currently recommended as a valid option for this approach by NCCN guidelines in high risk LACC, which is supported by data from the FOxTROT and OPTICAL trials [9, 10]. Current claims from literature that triplet chemotherapy (FOLFOXIRI) is superior to doublet therapy in NAC cannot be confirmed because of the lack of directly comparative RCTs [14, 19]. In that sense, the use of triplet chemotherapy remains to this day merely investigational.

Higher objective response rates with triplet regimen in colorectal cancer can provide a rationale for an investigational application to achieve a maximum cytoreduction and avoid MVR in selected patients with T4 bulky tumors invading adjacent structures (such as the retroperitoneum in our patient) [13, 19]. The triplet regimen has successfully converted the bulky cT4b in our patient into a T2 tumor, offering eligibility for a standard CME right hemicolectomy without the need for wide adjacent organ excision. These results align with similar ones reported in a small single‐arm study and case reports [Table 1.] [5, 6, 14, 20–22]. Tomingaga et al. reported significant tumor shrinkage, with a transverse colon tumor reducing in size from 18 cm to 5 cm [5]. Kanto et al.′s report, a significant downstaging to Stage 1 was achieved using bevacizumab and FOLFOXIRI [21]. Hata et al. have successfully achieved bladder‐preserving resection, while Naito et al.′s approach has spared the resection of liver/duodenal [20, 22].

**Table 1:** Summary of studies investigating neoadjuvant FOLFOXIRI in LACC.

Study	Regimen	Setting/site	Key outcomes	Grade 3–4 toxicity
Zhou 2025/2016 [14]	FOLFOXIRI	Single‐arm Phase II (*n* = 72), high risk LACC (T4)	100% R0; 87% TRG score 0–1	51%–56% Hematologic toxicities
McGillivray, 2021 [6]	FOLFOXIRI + panitumumab	Case report (descending colon; ATM mutated)	Pathological complete response (pCR)	Grade 1–2 rash
Tominaga, 2020 [5]	FOLFOXIRI + bevacizumab	Case (transverse colon; mass measuring 18 cm in diameter)	R0; tumor shrinkage to 5 cm	Grade 2 neutropenia
Naito, 2020 [20]	FOLFOXIRI + bevacizumab	Case (ascending colon invading liver and the duodenum)	PCR; resection of the hepatic and duodenal invasion	Grade 3 neutropenia
Kanto, 2020 [21]	FOLFOXIRI + bevacizumab	Case report (transverse colon with gastric invasion)	Pathology Stage I; significant downstaging	Grade 3 (neutropenia/anorexia)
Hata, 2022 [22]	FOLFOXIRI + bevacizumab	Case report (sigmoid colon with bladder invasion)	Partial response; bladder preserved	Not specified

This case supports the administration of intensive chemotherapy within resource‐limited settings. Countries facing severe economic sanctions or fragile healthcare infrastructures—such as Syria—have great difficulties accessing routine advanced molecular testing (MMR IHC, RAS/BRAF NGS) because of failure of supply chains and reagent shortages [23]. The choice of triplet chemotherapy as a neoadjuvant approach was supported by the availability of generic cytotoxic drugs (5‐FU, Oxaliplatin, Irinotecan), which are provided through national public health procurement programs, and the fact that 85% of sporadic colon cancer cases are pMMR chemo‐sensitive tumors [12]. Nonetheless, this intensified cytotoxic neoadjuvant approach can be considered for carefully selected fit patients (ECOG 0). Treatment toxicities such as hematologic (neutropenia) and anorexia are more frequent with FOLFOXIRI [14]. Our case indicates that this regimen can be managed safely in provided healthcare infrastructure using generic G‐CSF such as filgrastim, standard antiemetic protocols, and despite short treatment interruptions, without affecting the surgical outcomes.

Compared with our case, NAC FOLFOXIRI has been evaluated in small, prospective, single‐arm trials (Zhou et al.). However, this report offers unique value in demonstrating real‐world pheasant and clinical decision‐making in sanctions‐affected and resource‐limited healthcare infrastructures. In addition, our case highlights that traditional clinical and radiological risk stratification can guide intensive therapy to achieve satisfactory results.

On the other hand, this report has several limitations. Results from one patient are observational and cannot generate hypotheses for valid testing to conclude which is superior between doublet and triplet therapies. In addition, posttreatment surveillance is currently limited to a relatively short duration of 1.5 years (18 months) in comparison with durations of 3 and 5 years in order to evaluate disease‐free and overall survival benefits. Moreover, the absence of molecular tastings prevents the ability to establish correlation between tumor genomic profile and clinical response.

In conclusion, NAC FOLFOXIRI can achieve significant volumetric tumor downstaging and enhance the probability to achieve R0 resection rates, albeit in selective high‐risk LACC cases. While requiring close monitoring and management of treatment toxicities, these findings provide hypotheses‐generating evidence pending results from ongoing RCTs such as the FOxTROT3, which will compare the doublet and triplet NAC in terms of efficacy and toxicity [15].

**Nomenclature**
AJCC: American Joint Committee on Cancer
ATM: ataxia‐telangiectasia mutated
BRAF: B‐rapidly accelerated fibrosarcoma
CA19‐9: cancer antigen 19‐9
CEA: carcinoembryonic antigen
CME: complete mesocolic excision
ECOG: Eastern Cooperative Oncology Group
EGFR: epidermal growth factor receptor
G‐CSF: granulocyte colony stimulating factor
IHC: immunohistochemistry
KRAS: Kristen rat sarcoma
LACC: locally advanced colon cancer
MMR: mismatch repair
MVR: multivisceral resection
NAC: neoadjuvant chemotherapy
NCCN: National Comprehensive Cancer Network
NGS: next generation sequencing
NRAS: neuroblastoma rat sarcoma
pCR: pathological complete response
R0: complete resection
RCTs: randomization control trials

## Author Contributions

Dr. Osama Alazka: conceptualization, investigation, writing—original draft, software, writing—review and editing. Dr. Rita Mohammad: conceptualization, writing—original draft, writing—review and editing, methodology. Dr. Haya Alreem Saied: resources, writing—original draft. Dr. Hamza Abedallaa: writing—original draft, methodology, conceptualization, writing—review and editing. Dr. Nader Abedallaa: conceptualization, investigation, methodology, writing—review and editing, supervision, and resources.

## Funding

No funding was received for this manuscript.

## Consent

Informed consent was obtained for the publication of deidentified information and case details.

## Conflicts of Interest

The authors declare no conflicts of interest.

## Supporting information


**Supporting Information** Additional supporting information can be found online in the Supporting Information section. File S1:CARE checklist of information to include when writing a case report.

## Data Availability

The data that supports the findings of this study are available from the corresponding author upon request.
